# Mapping of the spontaneous deletion in the *Ap3d1* gene of mocha mice: fast and reliable genotyping

**DOI:** 10.1186/1756-0500-1-119

**Published:** 2008-11-25

**Authors:** Kim Ryun Drasbek, Mai Marie Holm, Marion Delenclos, Kimmo Jensen

**Affiliations:** 1 Synaptic Physiology Laboratory, Department of Physiology and Biophysics, Aarhus University, Ole Worms Allé Bldg 1185, DK-8000 Aarhus C, Denmark; 2 Department of Molecular Biology, Aarhus University, Gustav Wieds Vej 10 C, DK-8000 Aarhus C, Denmark

## Abstract

**Background:**

The *mocha *mouse carries a spontaneous deletion in the *Ap3d1 *gene, encoding the delta 1 subunit of the adaptor related protein complex 3, (Ap3d1), and subsequently lack the expression of functional AP-3. This leads to a deficiency in vesicle transport and storage, which affects neurotransmitter vesicle turnover and release in the central nervous system. Since the genomic sequence of the *Ap3d1 *gene of *mocha *mouse is not known, precise mapping of the deletion as well as reliable genotyping protocols are lacking.

**Findings:**

We sequenced the *Ap3d1 *gene (HGNC GeneID: 8943) around the deletion site in the *mocha *mouse and revealed a 10639 bp deletion covering exon 2 to 6. Subsequently, new PCR primers were designed yielding a reliable genotyping protocol of both newborn and adult tissue. To examine the genotypes further, hippocampal neurons were cultured from *mocha *and control mice. Patch-clamp recordings showed that *mocha *neurons had a higher input resistance, and that autaptic EPSC in *mocha *cultures depressed faster and stronger as compared with control cultures.

**Conclusion:**

Our study reports the sequence of the deleted part of the *Ap3d1 *gene in *mocha *mice, as well as a reliable PCR-based genotyping protocol. We cultured hippocampal neurons from control and *mocha *mice, and found a difference in input resistance of the neurons, and in the synaptic short-term plasticity of glutamatergic autapses showing a larger synaptic depression than controls. The described procedures may be useful for the future utilization of the *mocha *mouse as a model of defective vesicle biogenesis. Importantly, as genotyping by eye color is complicated in newborn mice, the designed protocol is so fast and reliable that newborn mice could rapidly be genotyped and hippocampal neurons dissociated and cultured, which is normally best done at P0-P2.

## Background

For the study of neuronal function, a range of useful mouse models exist where spontaneous genomic alterations have occurred. In order to efficiently utilize these models, a precise insight into the altered sequence must be available, and reliable genotyping protocols must be set up [1].

Adaptor related proteins (APs) are a group of heterotetrameric complexes thought to be involved in endocytosis and vesicle biogenesis in neuronal and non-neuronal tissues [2]. Of the four adaptor related proteins described so far (AP-1 to AP-4), AP-3 is expressed in brain and preferentially located in endosomal membranes. AP-3 is composed of a δ, β, μ and ρ subunit, all thought to be necessary for AP-3 to participate in late endocytotic steps and vesicle budding from endosomes. Therefore, it has been speculated that AP-3 participates in synaptic vesicle biogenesis and thereby in the regulation of synaptic transmission in the CNS. Indeed, two recent studies have found that fast synaptic transmission is impaired in AP-3 deficient neurons [3,4].

The *mocha *mouse carries a deletion in the AP-3 δ subunit and homozygous *mocha *mice display epileptiform activity [5], auditory changes [6], pigmentation dysfunction, and storage deficiency in platelets [6], while heterozygotes are phenotypically similar to wild types. It is possible that the CNS phenotype is caused by impaired synaptic vesicle formation and recycling. As pigmentation is affected by the mutation, genotyping of *Ap3d1 *deficient *mocha *mice may be done by inspection of eye color due to different degrees of eye pigmentation [7]. However, this procedure is challenging, especially in younger animals, and not completely reliable.

The exact sequence of the altered gene in *mocha *mice is unknown. Therefore, we have sequenced the genomic deletion and used this to design an efficient genotyping protocol, which facilitated culturing and electrophysiological studies of primary *mocha *neurons.

## Results

### Sequencing of the gene deletion in mocha mice and developing a new genotyping protocol

**Figure 1:**
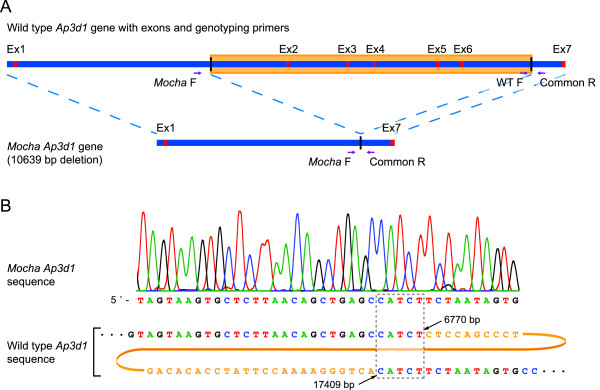
**Position of the deletion in the *Ap3d1 *gene in *mocha *mice**. A) Outline of the *Ap3d1 *gene with exons (red) and introns (blue). The deletion in the *mocha *mice is illustrated by the orange box in the wild type *Ap3d1 *gene. The position of the genotyping primers are shown on both the WT and *mocha *genes. B) Sequencing of the *mocha Ap3d1 *gene reveals the deletion site, which shares a CATCT motif in both the upstream and downstream sequence. Alignment of the *mocha Ap3d1 *gene with the WT gene shows that the mutated *Ap3d1 *gene contains the first 1–6770 bp and the last 17409–18539 bp. The dashed box illustrates the position of the repeated CATCT sequence. Nucleotides only present in the WT sequence, and deleted in *mocha *mice, are colored orange.

Since exons 2–6 were previously found to be deleted in the *mocha *mouse [6], primers were designed in intron 1 and 6 to obtain an overview of the deletion. In addition to primers available in the literature, several new primers were designed possibly flanking the deletion site (Table 1). Genomic DNA was purified using the fast HotShot DNA purification method [8] from adult *mocha*, heterozygote and WT mice as determined by eye and coat color. PCRs using different combinations of the three forward (F) and three reverse (R) primers (Table 1) were performed. As expected, no PCR products were seen when using WT genomic DNA as template possibly due to the large products (7700–12800 bp) and the harsh DNA extraction method used. In contrast, using *mocha *genomic DNA, PCR products were generated using the *Mocha *F1, Intron1 F, Intron7 R and *Mocha *R2 primers. No products were seen when including either the *Mocha *F2 or *Mocha *R1 primers, indicating that these two primers are situated within the deletion. DNA sequencing and subsequent sequence analysis of a 1.3 kb PCR product revealed a short DNA motif (CATCT) found in both intron 1 ending at 6770 bp and in intron 6 starting at 17409 bp, giving rise to a 10639 bp deletion (Fig. 1).

**Table 1:** Primers used for sequencing and genotyping

**Primer**	**Sequence**	**Start**	**End**
*Mocha *F1	5'- CCT CAA GCC CGT CAG AAG- 3'	5322	5339
Intron1 F	5'- TTG AAC TCG GAG ACA TCC TCT GC- 3'	5616	5638
*Mocha *F2	5'- CTG GGT GGC ATT TAG TTA CTT GAG GC- 3'	7790	7815
*Mocha *R1	5'- CTT CTG GGC TGG CTG GAT GTA AG- 3'	15485	15507
Intron7 R	5'- CCA ATA CCA ACC AAC CAA CAA CC- 3'	17473	17495
*Mocha *R2	5'- TGA CAT TAA CAG CCG CAG AC- 3'	18391	18410
**Genotyping primers**			
*Mocha *F	5'- CCT GCT TCT GAG TGC TTG GG- 3'	6193	6212
WT F	5'- GGC TGC TTC TGG ACT GTT CG- 3'	17006	17025
Common R	5'- CTT CTC CAC CCT CTT TCC ACC C- 3'	17661	17682

List of the forward (F) and reverse (R) primers used for the sequencing of the deletion and for the developed genotyping protocol (the last three primers). "Start" and "End" numbering corresponds to the full length sequence of the *Ap3d1 *gene.

**Figure 2:**
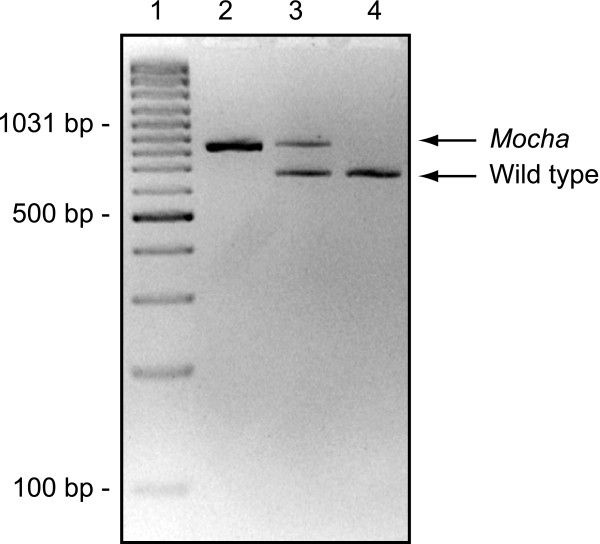
**Genotyping of *mocha *mice**. Agarose gel electrophoresis of a genotyping-PCR. Using the primers described in table 1, *mocha *mice resulted in an 847 bp PCR product and wild types generated a 677 bp product while both bands were clearly evident in heterozygotes, as expected. Lane 1: GeneRuler 100 bp Plus DNA ladder (Fermentas, Vilnius, Lithuania), lane 2: *mocha *homozygote (847 bp) (*Ap3d1 *-/-), lane 3: heterozygote (*Ap3d1 *+/-), lane 4: AP-3 wild type (677 bp) (*Ap3d1 *+/+). The genotyping protocol worked well in both newborn and adult mice.

For the genotyping protocol, primers were designed (Fig. 1, Table 1) sharing the downstream primer, while having specific upstream primers for *mocha *and WT, respectively (Fig. 2).

### Cultured hippocampal neurons from mocha mice show increased input resistance

To analyze for functional differences, microcultures of hippocampal neurons from genotyped newborn control and *mocha *mice were prepared. Cell capacitance was measured electrophysiologically, showing no significant differences (22.4 ± 1.4 vs. 19.4 ± 1.4 pF, *P *= 0.12) between control (*n *= 18) and *mocha *neurons (*n *= 21), indicating equal cell size.

**Figure 3:**
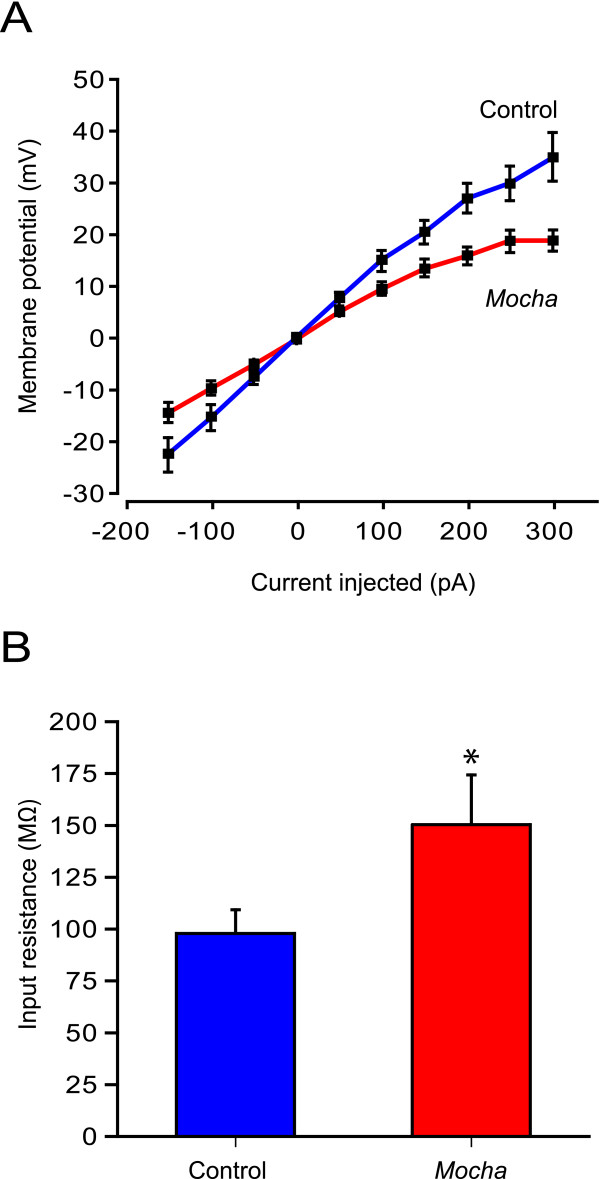
**Cultured hippocampal *mocha *neurons show higher input resistance**. A) The passive membrane current-voltage relationship was examined in control and *mocha *neurons in primary hippocampal culture. In current clamp, positive and negative current injections were made and the membrane potential deflections were measured. The holding potential before current was injected was -60 mV. The graphs result from recordings in control (*n *= 11) and *mocha *neurons (*n *= 13). B) The neuronal input resistances were calculated from the slope of the linear part of the current-voltage curves shown in A). *Mocha *neurons had higher input resistances, indicating a smaller membrane conductance compared with controls. *: *P *< 0.05 unpaired *t*-test. Values are expressed as mean ± SEM.

Neurons were then current-clamped and held at -60 mV while positive and negative currents (-150 to +300 pA) were subsequently injected to obtain a current-voltage relationship (Fig. 3A). From these curves, the input resistance was estimated using linear regression of the linear part (-150 to +100 pA) for each cell. The input resistance was significantly increased in *mocha *neurons (150.3 ± 22.1 MΩ, *n *= 13) compared with controls (97.8 ± 12.4 MΩ, *n *= 11, *P *< 0.05, Fig. 3B). This suggests that the membrane conductance is smaller in *mocha *neurons, indicating reduced surface expression of ion channels.

### Hippocampal mocha neurons show increased short-term synaptic depression

**Figure 4:**
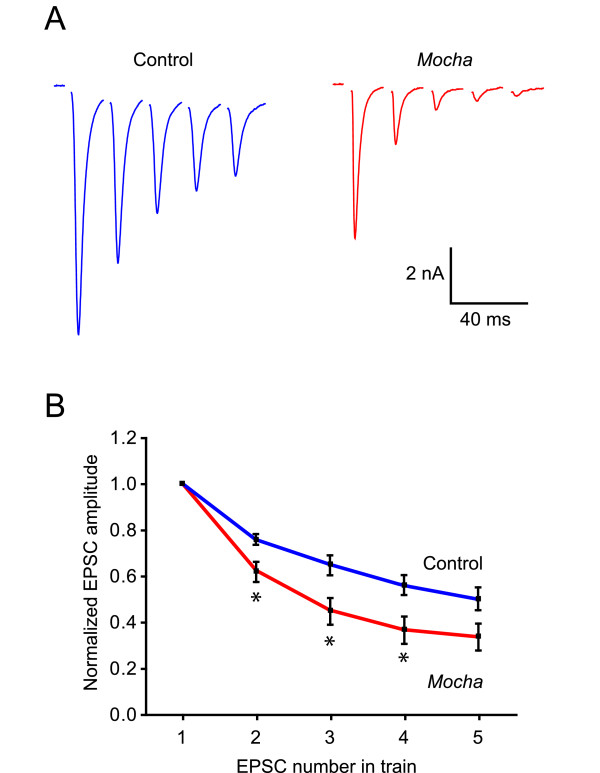
***Mocha *synapses depress faster and to a greater extent**. A) Autaptic EPSCs were evoked at 50 Hz in control and *mocha *mice. B) Normalized EPSC amplitude as a function of the EPSC number in the train. It is seen that the *mocha *(*n *= 9) synapses depress more than control (*n *= 7) (*: *P *< 0.05). Un-paired *t*-tests were used to statistically compare relative EPSC amplitude from control and *mocha *neurons, and values are expressed as mean ± SEM.

Since AP-3 has recently been shown to be involved in short-term synaptic plasticity [4], we tested whether any apparent changes in regulation of transmitter release could also be seen in *mocha *neurons. On microislands, the axon of the neuron forms autapses onto itself and a postsynaptic current can be evoked in the same cell. Since the vast majority of cells are glutamatergic neurons [9], synaptic activity leads to an excitatory postsynaptic current (EPSCs) in the cell. Neurons were voltage-clamped at -70 mV and the holding potential was stepped to +20 mV for 2–3 ms, evoking an unclamped action-potential that propagated orthodromically to the boutons. These single evoked EPSC could be blocked completely by 10 μM CNQX (6-cyano-7-nitroquinoxaline-2,3-dione, a competitive AMPA and kainate receptor antagonist) and their amplitudes reached on average 3.5 ± 0.89 nA (*n *= 11) in control and 2.1 ± 0.67 nA (*n *= 12, *P *> 0.05) in *mocha *cultures. When EPSCs were evoked by brief high-frequency trains of 5 pulses delivered at 50 Hz (Fig. 4A), the EPSCs showed a significantly stronger synaptic depression in *mocha *than in control neurons. The largest difference in synaptic depression was seen at pulse 3, where the EPSC amplitudes (normalized to the first EPSC) were reduced to 65 ± 3.8% in control (*n *= 7) versus 45 ± 5.8% in *mocha *(*n *= 9, *P *< 0.05). At pulse 4, the EPSCs were depressed to 56 ± 4.3% in control versus 37 ± 5.9% in *mocha *(*P *< 0.05, Fig. 4B). This corroborates that the AP-3 complex may participate in the turnover and/or exocytosis of small neurotransmitter vesicles [10-12] also in cultured neurons.

## Discussion

This study had three goals namely 1) to sequence the genomic deletion in *mocha *mice, 2) to set up a reliable PCR genotyping protocol for unambiguous identification of genotypes of all offspring from heterozygous breeding, and 3) to perform initial functional studies in cultured *mocha *neurons of membrane properties and synaptic plasticity. Reaching all three goals, the protocol reported here will facilitate further functional studies of *mocha *neuronal and non-neuronal cells in a variety of settings, including cell culture.

### The sequence of the genomic deletion in mocha mice

As the heterotetrameric AP-3 protein complexes are known to be involved in vesicle sorting and fusion, it is comprehensible that AP-3 deficiency leads to vesicle storage and transport deficiencies in various tissues [6]. While AP-3 is composed of δ, β3, μ3 and ρ3 subunits, the *mocha *mutation affects the δ subunit of AP-3, leading to a lack of expression of the whole complex. Although mice deficient for each individual subunit exist, the *mocha *mutation leads to the most severe phenotype, which has been explained by the finding that the δ subunit is common to all AP-3 complexes [6].

Since the exact sequence of the genomic deletion of *mocha *mice was previously not known, we sequenced the *mocha *gene and found that a 10639 bp long segment of the *Ap3d1 *gene was deleted including exons 2–6. Interestingly, we found that the site of deletion was flanked by a repeated sequence 5'CATCT (Fig. 1) in the wild type sequence. Genomic deletions can be the result of recombination between SINE repeat elements, however, as these elements usually are 2–300 bp long with a high homology between the recombining repeats, this is probably not the cause of the *mocha *deletion, since only the CATCT sequence is repeated. Therefore, we speculate that the spontaneous deletion has arised possibly by some kind of "replication slip" of the DNA polymerase from one repeat to the other repeated sequence most likely during replication of the genomic DNA. Afterwards, the deletion has been conserved since heterozygous and to some degree homozygous mice are able to reproduce.

### Functional alterations in cultured hippocampal neurons of mocha mice

Patch-clamp recordings showed that the *mocha *neurons have a larger input resistance. A likely explanation for this is a decrease in membrane conductance associated with a reduced surface expression of functional ion channels. Since AP-3 is known to be involved in the transport and targeting of ion channels, such as the chloride channel ClC-3 [13], it is possible that the cultured *mocha *neurons display a certain degree of missorting of ion channels. The exact nature and extent of such an ion channel missorting may be the topic for further investigations.

Although there are still no functional data to support this, it is possible that an increased membrane resistance would make the *mocha *neurons more responsive to excitatory synaptic input, which could in fact make them hyperexcitable. Noting that *mocha *mice display an epileptiform phenotype [5], it could be interesting to investigate whether the increased input resistance also exists *in vivo*.

The other finding of our paper was that glutamatergic autapses on cultured *mocha *neurons display a stronger synaptic depression of EPSCs in response to brief stimulus trains. Synaptic depression usually depends on depletion of the readily releasable pool of synaptic vesicles and on the refilling of that pool. Thus, the Ap3d1 deficiency appears to lead to alterations in the exocytosis and possibly turnover of synaptic vesicles in cultured neurons. Short-term synaptic plasticity of excitatory neurotransmission has recently been studied by others, who found that field EPSPs (excitatory postsynaptic potentials) actually depressed *less *in adult *mocha *mouse brain slices in response to repetitive stimulation [4]. This could be due to differences in vesicle turnover and release probability at excitatory synapses between adult slices and postnatal hippocampal cultures. While excitatory synapses in culture usually display a higher release probability and show a substantial synaptic depression [9], it is likely that their synaptic vesicle endo- and exocytotic processes are governed by the development stage and culture conditions. Despite this, hippocampal cultures may be very useful in future studies of the involvement of Ap3d1 in vesicle release using combinations of patch-clamp and imaging techniques, such as FM1-43 or SynaptopHluorin signals, that can report both vesicle endo-and exocytosis [14]. Since AP-3 may both participate in the synchronous [4] and asynchronous release of neurotransmitter [12], and controls the targeting of other presynaptic proteins, such as TI-VAMP (tetanus neurotoxin-insensitive vesicle-associated membrane protein) [3,12], Vglut1 (vesicular glutamate transporter 1) [15] and VGAT (vesicular GABA transporter) [16], the deletion in the *Ap3d1 *gene of *mocha *mice is likely to have an impact on the function of many different synapses.

## Methods

### Mouse supplier and breeding

Mice were obtained from The Jackson Laboratory (Bar Harbor, Maine, USA) strain name: STOCK *gr *+/+ *Ap3d1*^*mh*^/J, stock number: 000279 and kept in a university facility on a 12/12 hours light/dark schedule with unlimited access to food and water. All animal procedures were performed according to the European Communities Council Directive of 24 November 1986 (86/609/EEC).

### HotShot genomic DNA extraction

A modified HotShot protocol [8] was used to purify DNA. 50 μl of alkaline lysis buffer (25 mM NaOH, 0.2 mM Na-EDTA, pH 12.3) was added to a 2 mm clip of the mouse tail on ice. Lysis was carried out in a PCR machine (Peltier Thermal Cycler, PTC-200, MJ Research, Waltham, MA, USA) for 30 min at 65°C followed by 16 min at 98°C. Afterwards the samples were neutralized by adding 50 μl of a neutralizing buffer (40 mM Tris-HCl, pH 5.0) on ice.

### PCR for the mapping of the deletion

2 μl purified DNA (diluted 1:5 in TE buffer after HotShot) was used for the PCR performed in 25 μl consisting of 3.5 mM MgCl_2_, 0.3 mM dNTP, 0.5 μM of forward and reverse primer (Table 1) and 0.05 units/μl of HotStarTaq polymerase (Qiagen, Hilden, Germany). The PCR was performed as follows: The HotStarTaq enzyme was activated at 95°C for 15 min followed by 30 cycles of a 3-step protocol (95°C for 30 sec, 60°C for 30 sec and 72°C for 10 min) and a final elongation step at 72°C for 10 min. The reaction was visualized on a 2% agarose gel in TBE buffer.

All DNA sequencing was done by Eurofins MWG Operon (Ebersberg, Germany) and analyzed using VectorNTI (Invitrogen, Paisley, United Kingdom).

### Genotyping protocol

The PCR was performed as above with the following changes: 0.5 μM of primer "Common R", 0.5 μM of primer "WT F", 0.5 μM of primer "*Mocha *F" was used together with a shorter elongation step in each cycle at 72°C for 1 min. The reaction was visualized on a 2% agarose gel in TBE buffer.

### Hippocampal cell culture

Single hippocampal neurons from newborn (P1-P2) mice were cultured on microislands, using an established protocol [9]. Briefly, coverslips with stamps of ~200 micrometer large collagen islands covered with Poly-D-lysine and a feeder layer of astrocytes were prepared [9]. Brains were dissected out, meninges and vascular tissue washed off and hippocampi were enzymatically dissociated in papain in DMEM for 1 hour at 37°C and plated at low density on microislands. Neurons were allowed to mature for 14–21 days before recording, and only microislands containing a single neuron were used.

### Electrophysiology

Current- or voltage-clamp whole-cell patch-clamp recordings were made using 3–4 MΩ patch-pipettes (P-97 puller, Sutter Instrument Co, Novato, CA, USA) and filled with (in mM): 135 K-Gluconate, 10 HEPES, 1 EGTA, 4.6 MgCl_2_, 4 Na-ATP, 15 creatine phosphate, 50 U/ml phosphocreatine kinase (295 mOsm kg^-1^, pH 7.3). The extracellular solution contained (in mM); 140 NaCl, 2.4 KCl, 10 HEPES, 10 glucose, 4 CaCl_2_, and 4 MgCl_2 _(300 mOsm, pH 7.4). Input resistance was estimated in current-clamp by injecting 100 ms long current steps and measuring the resulting voltage deflection. To record autaptic EPSCs, neurons were voltage-clamped at -70 mV using an EPC-10 amplifier (HEKA Elektronik, Lambrecht, Germany) and stimulated by stepping the membrane potential to 0 mV for 3 ms. This evoked a single break-away action-potential that propagated to the autaptic nerve terminals and gave rise to a short latency EPSC in the same cell. Series resistances were below 15 MΩ and compensated by 70–80%. Currents were low-pass filtered at 5 kHz and acquired at 10 kHz using Pulse (HEKA).

## Competing interests

The authors declare that they have no competing interests.

## Authors' contributions

KRD planned and executed the experiments as well as outlined the artwork and wrote the article. MMH analyzed the results, prepared the artwork, and wrote the manuscript. MD analyzed data and prepared the article. KJ planned and coordinated the project, and prepared the article. All authors approved the final manuscript.
